# LGBTQ+ Adult Sexual Violence Critical Scoping Review: Victimization Risk Factors

**DOI:** 10.1177/15248380241311930

**Published:** 2025-01-22

**Authors:** Jessica Ison, Sophie Hindes, Bianca Fileborn

**Affiliations:** 1 La Trobe Rural Health School, La Trobe University, Melbourne, VIC, Australia; 2 Australian Research Centre in Sex, Health and Society, La Trobe University, Melbourne, VIC, Australia; 3 Criminology, School of Social and Political Sciences, The University of Melbourne, Melbourne, VIC, Australia

**Keywords:** sexual violence, LGBTQ+, queer, transgender, victimization

## Abstract

Sexual violence experienced by LGBTQ+ adults is a rapidly expanding field of academic study. Therefore, there is a need for a synthesis and critical analysis of the research. The aim of this review was to conduct a critical review of the academic literature on adult LGBTQ+ sexual violence and to provide recommendations for future research. A total of 10,845 papers were identified through a comprehensive scoping review approach and 108 met the criteria for inclusion. The findings are reported across two papers. This second paper reports on the risk factors for victimization: alcohol and other drugs (AOD); homophobia, biphobia, transphobia, and minority stress; “risky” sexual behavior and HIV transmission; and child sexual abuse (CSA). AOD use was seen as a risk factor for sexual violence, yet the causal relationship was not always clear. Minority stress was conceived of as an individual issue with undertones of victim-blaming. “Risky” sexual behavior research, at times, framed LGBTQ+ sex as “risky” and failed to account for the specific needs of LGBTQ+ communities. Research on adult sexual violence risks had a focus on CSA that often neglected broader structural issues. In general, across the studies, there was a focus on individual-level research that analyzed survivor behavior, resulting in victim blaming. This paper advocates for expanding LGBTQ+ sexual violence research beyond just individual risk factors, shifting research away from constructing LGBTQ+ people as “risky,” and expanding research to include a more intersectional analysis that goes beyond heteronormative and cisnormative inquiry.

## Introduction

Sexual violence has received increased focus as an area of study over the past few decades, with feminist efforts demonstrating the pervasive nature of sexual violence globally. The increased attention on sexual violence is due to the important research and advocacy of feminists, particularly since the 1980s (Loney-Howes, 2019). While feminist research has been vitally important in bringing attention to the extent and harms of sexual violence, research to date has focused predominantly on cisgender heterosexual women victim-survivors and cisgender heterosexual men as perpetrators (Fileborn, 2012; Mortimer et al., 2019). This has meant that understandings of sexual violence have become synonymous with critiques of heterosexuality and heterosexual gendered power dynamics, with a limited understanding of the unique factors that shape LGBTQ+ people’s experiences (Hindes et al., 2025).

The heteronormative framing of sexual violence is, however, beginning to shift with an increasing body of research broadening the focus to include the experiences of diverse communities, including LGBTQ+ communities (e.g., Alessi et al., 2021; Klein et al., 2023). However, to date, no scoping review has drawn together quantitative and qualitative English language research on LGBTQ+ adult (ages 14+) sexual violence. We aimed to rectify this gap through a critical scoping review of the academic peer-reviewed literature to understand the state of the field and identify gaps and areas for improvement. Due to the large number of studies, we have addressed the findings in two publications. Our first paper presents the findings relating to victimization and perpetration prevalence (Hindes et al., 2025). As we outline in that paper, we found that LGBTQ+ people experience high rates of sexual violence, with rates the highest for bisexual women and trans and gender-diverse people. We also found that while studies on perpetration are limited, most perpetrators of sexual violence are men for victim-survivors of all genders and sexualities. However, we also identified significant concerns with the existing research, including methodological limitations in sexual violence prevalence and gender and sexuality measures, collapsing of diverse LGBTQ+ samples into limiting categories (e.g., sexual minority), a lack of research on trans and gender-diverse people, and an oversaturation of U.S.-based studies and university/college student samples (Hindes et al., 2025). In this paper, we assess the state of knowledge of the risk factors associated with LGBTQ+ sexual violence. We identified four risk factors that warranted critical attention: alcohol and other drug (AOD) use; homophobia, biphobia, transphobia, and minority stress; “risky” sexual behavior and HIV transmission; and child sexual abuse (CSA). In the following sections, we briefly outline the literature relating to these themes before detailing the scoping review methodology, results, and discussion.

### Sexual Violence Risk Factors

Empirical research on sexual violence has sought to understand the risk factors associated with sexual violence victimization and, to a much lesser extent, perpetration. Due to the limited research on risk factors for perpetration, we focus on victimization. By risk factors, we mean the behaviors or contexts that are measured and compared against victimization prevalence to examine whether these factors are associated with increased rates of sexual victimization. Much of this literature stems from public health disciplines, which are seeking not only to reduce the mental and physical health consequences for victim-survivors, but also the “economic burden” of sexual violence (Vivolo et al., 2010, p. 1811). We use the language of risk factors as this is the language most commonly used across the field. However, this framing tends to prioritize individual behavior change over addressing structural causes, which is an issue we challenge throughout this paper.

In studies of heterosexual and cisgender populations, one of the most common risk factors for adult sexual violence has consistently been shown to be previous experiences of CSA (Basile & Smith, 2011; Scoglio et al., 2021). As Scoglio et al.’s review of the literature found, women who have experienced CSA are 2–13 times more likely to experience victimization in adulthood. Several explanations for this have been offered, including that those who experience CSA are more likely to engage in “risky” sexual behaviors, have post-traumatic stress disorder (PTSD), experience emotional dysregulation, and engage in other maladaptive coping strategies such as “problem drinking and substance use”—all of which are associated with revictimization (Scoglio et al., 2021). Revictimization literature has often narrowly focused on these individual factors shaping revictimization, without looking at broader contexts such as victim-blaming or structural disadvantage (Corbett et al., 2025).

Apart from CSA, studies on victimization risk tend to disproportionately focus on women’s individual-level behavioral risk factors, including AOD consumption (see, e.g., Crawford et al., 2008; Krebs et al., 2009; Testa & Livingston, 2018) and what some researchers term “risky” sexual behavior including having more sexual partners, engaging in “hook up” culture, casual sex, sex with multiple partners, inconsistent use of contraception, and engaging in “early” intercourse (see, e.g., Testa et al., 2010; Turchik & Hassija, 2014). These researchers argue that engaging in these behaviors increases women’s risk of sexual victimization. However, this focus lends itself to victim-blaming attitudes and can put the onus of sexual violence prevention onto victim-survivors, particularly in the context of alcohol and other drugs use (Ison et al., 2024). For example, Crawford et al.’s (2008, pp. 270–271) study of “alcohol- or other drug enabled and drug facilitated sexual assault” concluded that college women who are victims “make riskier behavioural choices compared to nonvictims” and therefore “victims of sexual assault may benefit from programs with a primary focus on the factors that place them at risk for sexual assault.” As Bonar et al.’s (2022) review of prevention strategies for sexual violence among college students found, community-level risk factors have been understudied, and there is a greater need to address institutional and structural factors. Porat et al.’s (2024) scoping review of sexual violence prevention interventions also reiterates that the focus has been on individualized education programs, which show limited effect. However, across the field of heterosexual sexual violence research, this victimization risk focus is somewhat balanced by a significant body of research on the risk factors for perpetration (see, e.g., Porta et al., 2017, and Tharp et al., 2012, for overviews of the literature).

Sexual violence theorists have emphasized the need to understand the social and cultural drivers of sexual violence. For example, Gavey (2005) theorizes how sexual violence becomes reframed as “just sex” through cultural and social representations of heterosexual sex that constructs men’s behavior as normal and natural. However, as we found in our first article, research on perpetration against LGBTQ+ people is extremely limited (Hindes et al., 2025), with a significant focus on victimization risk factors for LGBTQ+ adult victim-survivors across the field. Therefore, in this paper, we critically synthesize the research on victimization risk factors for LGBTQ+ people.

### The Current Study

While there has been important research on the risk factors associated with sexual violence victimization, it has often failed to consider LGBTQ+ people. Across both papers, we have drawn together both qualitative and quantitative peer-reviewed studies published in English on LGBTQ+ sexual violence, using a scoping review methodology to fill this gap, which, to our knowledge, no other review has done. Across both articles, we critically reflect on the current research on LGBTQ+ adult sexual violence. In the first publication, we reported the prevalence of both victimization and perpetration. In this article, we focus on the research relating to risk factors for victimization. We focus on victimization, as there were very few papers in our study that looked at perpetration in any detail, and even fewer had information about the risk factors for perpetration. We also focus on adults, where adulthood is variably considered to commence at age 14 to 18 across studies. In paper one, we offer a significant critique of how sexual violence is measured across adulthood and across the lifetime, and how disparities in this measurement make it difficult to compare studies. We first briefly outline the methodological approach, before presenting key findings. Our discussion then offers critical reflections on the state of the field, and considers how research on LGBTQ+ sexual violence can be expanded and improved.

Before continuing, we have a note on terminology. Throughout, LGBTQ+ is used as an umbrella term which aims to be as inclusive as possible of sexuality and gender-diverse people. However, as our studies range from 1999 to 2021, terminology differs across the studies. Some terminology may now be dated, yet when discussing individual studies we have chosen to use the terminology from the original studies to ensure that we adhere to their original context and meanings. This includes the labels individual studies used to describe sex, gender, and sexuality. We note that because terminology changes, what was once acceptable can become stigmatizing or problematic. For example, terms like homosexual can now be considered stigmatizing (American Psychological Association, 2022). Also, while there are differing opinions from within intersex communities, there is strong advocacy for people with intersex variations to be addressed independently from the LGBTQ+ umbrella (Carpenter, 2023). We, therefore, have not included people with intersex variations and signal this as an area of further study. Additionally, while many studies report on “men,” “women,” “males,” and “females,” they often have not specifically asked about trans or gender-diverse identity. While it is possible that transgender individuals participated in these studies, unless transgender people were explicitly included as a category of analysis, it is impossible to know with any confidence whether a given study included transgender participants or was rather composed of a wholly cisgender sample. If transgender participants were included but collapsed into the categories of men/male or women/female, we argue that such a decision effectively renders transgender participants as invisible in the research. As we point out across both papers, this is a concerning limitation, which underscores the need for more precise reporting on the inclusion of diverse gender identities in future studies.

## Methods

As noted, this is the second paper reporting on the findings of this critical scoping review. We have reported the method in depth in the first publication (Hindes et al., 2025). Here, we report the methods in brief. We used a scoping review methodology following Levac et al. (2010), which built on Arksey and O’Malley’s (2005) six-stage framework. We used a Preferred Reporting Items for Systematic Reviews and Meta-Analyses framework, which is provided in article 1. Studies were included if they met these inclusion criteria, which were developed to reduce the scope of the review while capturing papers that had a primary focus on LGBTQ+ populations and sexual violence:

• 50% or more of the paper about sexual violence (to capture studies where sexual violence was the focus of analysis, not an “add on” which is often the case in, e.g., studies of domestic violence or HIV research).
• 50% or more of the paper relates to LGBTQ+ participants (not related to sample size but focus of analysis).
• Paper primarily focuses on adult (age 14+) experiences of victimization or perpetration.
• English-only peer-reviewed articles.

We excluded any studies that:

• Had less than 50% focus on sexual violence (e.g., studies that were primarily about domestic violence but had some data on sexual violence).
• Had less than 50% focus on LGBTQ+ experiences (e.g., studies that had some LGBTQ+ participants but LGBTQ+ sexual violence was not a primary focus of the article, or studies on same-sex sexual violence that was not LGBTQ+ specific, e.g., heterosexual “male rape”).
• Were primarily concerned with CSA, or where it was unclear whether participants were reporting on sexual violence that had occurred in childhood or adulthood (e.g., some “lifetime prevalence” studies).
• Were theses or dissertations.
• Were only theoretical or related to perceptions, rather than experiences of sexual violence victimization and perpetration.
• Were scoping and narrative reviews and non-peer-reviewed material such as conference papers, commentaries and editorials, books, and websites.
• Were focused exclusively on sexual harassment.

Our first search yielded 10,845 papers which we downloaded into Covidence (Veritas Health Innovation, 2022), where 4,375 duplicates were removed. Once titles and abstracts were screened, we had 230 papers remaining. Our final sample included 108 papers for extraction (see Supplemental Appendices A and B for a list of included studies) in Covidence. The results were then exported into a spreadsheet for analysis. All three authors then analyzed the studies overall and noted down the key themes emerging from the studies. The authors then met to discuss and map out their key themes. This was not an attempt to quantify findings. Rather, this is a critical scoping review where we critically interrogated the literature to draw out the most common themes which warranted interrogating. When mapping out the key themes, victimization prevalence studies dominated the results. Following this, all three authors independently found a similar focus on risk factors. We also identified other themes, such as impacts and help-seeking, but in light of existing literature reviews synthesizing this research (e.g., Klein et al., 2023; Zinzow et al., 2022), we decided to focus this paper on the underexplored topic of risk factors. The next step involved discussion and reflection, and a process of synthesizing the themes. Once in agreement, papers were then sorted into the themes. Due to the number of papers, we have reported the findings over two papers to ensure depth of analysis. This paper reports on the findings relating to victimization risk factors.

Studies were included in the themes if they made a significant contribution to the evidence on victimization risk factors. That is, they needed to go beyond just providing response rates to including some form of analysis. By way of example, many studies included a single measure on alcohol consumption that may have yielded only a simple percentage and no further discussion. For example, Martin et al. (2011) ask about whether respondents were incapacitated during the sexual assault, presenting this in tables but not providing analysis about the relationship between incapacitation and sexual assault. We also note that qualitative studies posed a significant barrier to this approach, as the participants may have discussed a topic such as alcohol or child abuse, but it was not the focus of the study. Thus, qualitative studies were included if the paper included a clear theme on the topic. However, many studies provided limited details of the study design and the findings, so some interpretation was needed. In this study, we only address risk factors for victimization, as this was overwhelmingly the subject of studies in our scoping review.

## Results

Across the studies, we identified a significant trend where LGBTQ+ sexual violence was researched only in relation to individuals, including their victimization risk factors (papers included in each theme are referenced in Table 1). While we are not saying that individual-level studies are wholly unwarranted, rather, as a field this means that the overarching findings focus on individual behavior as well as “solutions” to the problem being located within individual behavior change or risk management. We found that the most common risk factors studied across the literature were AODs, homophobia/transphobia/minority stress, “risky” sexual behavior, and CSA. We now move on to discuss each of these key themes.

**Table 1.** Risk factors by study.

Alcohol and other drugs	Aspin et al. (2009), Balsam et al. (2011), Beckman et al. (2018), Braun, Schmidt, et al. (2009), Connolly et al. (2021), DeLaney et al. (2020), Drückler et al. (2021), Gavey et al. (2009), Gilmore et al. (2014, 2021), Han et al. (2013), Hequembourg et al. (2011, 2013, 2015), Hughes et al. (2001, 2010), Jaffe et al. (2020), Johnson et al. (2016), Kalichman and Rompa (1995), Kalichman et al. (2001), Kelley, Ehlke, Braitman, and Stamates (2018), Kelley, Ehlke, Lewis, et al. (2018), López and Yeater (2021), Matsuzaka and David (2019), McGraw et al. (2020), McKie et al. (2020), Murchison et al. (2017), Peitzmeier et al. (2015), Ray et al. (2021), Rhew et al. (2017), Richardson et al. (2015), Semple et al. (2017), Siguvinsdottir and Ullman (2015), Snyder et al. (2018), Strike et al. (2001), and Wilkerson et al. (2021)
Homophobia/transphobia/minority stress	Beckman et al. (2018), Drückler et al. (2021), Flanders et al. (2020), Ford (2021), Gold et al. (2007, 2009), Gurung et al. (2017), Hequembourg et al. (2015), Hershow et al. (2021), Hickson and Davies (1994), Hughes et al. (2001), Jackson et al. (2017), Kammer-Kerwick et al. (2019), Krahé et al. (2013), Murchison et al. (2017), Nightingale (2021), Paquette et al. (2019), Sabidó et al. (2015), Salim et al. (2020), Solomon et al. (2021), Tilley et al. (2020), and Watson et al. (2021)
“Risky” sexual behavior	Braun, Terry, et al. (2009), Gaspar et al. (2021), Gavey et al. (2009), Hequembourg et al. (2013, 2015), Hershow et al. (2021), Jaffe et al. (2020), Kalichman and Rompa (1995), Kalichman et al. (2001), Kelley et al. (2018), Krahé and Berger (2001), Krahé et al. (2000), McGraw et al. (2020), Murchison et al. (2017), Ray et al. (2021), Sabidó et al. (2015), Semple et al. (2017), Shaw et al. (2012), Snyder et al. (2018), Tilley et al. (2020), Toro-Alfonso and Rodríguez-Madera (2004), Wells et al. (2016), and Wilson et al. (2017)
Child sexual abuse	Balsam et al. (2011), Beckman et al. (2018), Crump and Byers (2017), Fernández-Rouco et al. (2017), Gilmore et al. (2014), Gold et al. (2007, 2009), Han et al. (2013), Heidt et al. (2005), Hequembourg et al. (2011, 2013, 2015, 2021), Hughes et al. (2001, 2010), Jaffe et al. (2020), Kalichman et al. (2001), Krahé and Berger (2001), Lehavot et al. (2012), López and Yeater (2021), McGraw et al. (2020), Ratkalkar and Atkin-Plunk (2020), Ray et al. (2021), Semple et al. (2017), Sigurvinsdottir and Ullman (2016a, 2016b, 2015), Stoddard et al. (2009), and Toro-Alfonso and Rodríguez-Madera (2004)

### Alcohol and Other Drugs

Thirty-six studies included AOD as a sexual violence risk factor. In these papers, the focus was on either just alcohol (*n* = 17) or AODs (*n* = 19). Additionally, two focused on sexual practices that involved substances, popularly termed chemsex (with one of these also including AOD measures, thus the paper is included in both AOD and chemsex).

Studies approached AOD in two distinct ways: first as a factor in the sexual assault and second as a general question about drinking behavior. Only seven of the studies assessed AOD as a factor during the sexual assault. In these seven studies, research captured whether AOD facilitated the sexual assault such as the victim-survivor being intoxicated or incapacitated during the sexual assault. For example, Connolly et al. (2021) reported on the results of the Global Drug Survey. They aimed to report on the difference between transgender (*n* = 1,136) and cisgender (*n* = 74,277) respondents who had been taken advantage of while under the influence of AOD in the preceding 12 months and more than 12 months. They found that transgender people at both the 12 months and more than 12 months were more likely to be taken advantage of sexually than cisgender people when intoxicated. While the majority of these papers were on victimization, one qualitative study also included perspectives of people who used AOD to facilitate sexual assault (Strike et al., 2001).

While studies may have looked in general at the role of AOD in facilitating sexual assault, rarely did they include measures or questions relating to the specifics of AOD use in LGBTQ+ communities, with the exception of the two papers on chemsex (Drückler et al., 2021; Wilkerson et al., 2021). There were some examples outside chemsex, such as McKie et al.’s (2020) qualitative study with 350 gay, bisexual and other men who have sex with men (MSM), where participants talked about AOD as “blurring lines of consent” in sexual experiences as well as times where they were forced or pressured into using AOD during a sexual experience, particularly “poppers” or other sexual stimulants.

The majority of papers (*n* = 29) asked about the participants’ drinking in a specified timeframe such as the last 30 days, last year, or lifetime. Overwhelmingly, victim-survivors and LGBTQ+ victim-survivors were shown to have higher rates of AOD use. However, this needs to be taken with significant caution. Generally, the questions were basic measures of drinking behavior or they related to “drinking/taking substances to cope.” Yet, whether general questions or questions about coping mechanisms, the timeframe was often unclear and the questions rarely specified if they used substances *as a result* or *during* the sexual violence. Despite this, the results tended to associate sexual violence victimization and AOD use. For example, a respondent might have identified that they were a survivor since the age of 14, and then answer the AOD questions showing a high alcohol intake in the last week or month. The results may then highlight that LGBTQ+ victim-survivors had a higher alcohol intake but fail to account for the many and varied other aspects in the respondent’s life (see, e.g., Gilmore et al., 2021; Han et al., 2013).

In general, the only time where it was clear that experiences of sexual assault influenced increased alcohol consumption after an assault was in qualitative studies. For example, Matsuzaka and Koch (2019) conducted a study with 10 trans-feminine individuals. They found that participants had a range of health consequences due to sexual victimization, including substance use (*n* = 2). Outside of these qualitative studies, it was unclear whether victim-survivors’ higher AOD use was due to being victimized. Therefore, there is often a link drawn between AOD and sexual violence but it is unclear what relationship AOD has to sexual violence. We do not argue that there is not a relationship, rather, that caution needs to be used when generalizing these data particularly when talking about LGBTQ+ communities who are often already stigmatized for AOD use.

Further, while the relationship between AOD use and a history of sexual violence is unclear, the framing of the questions means that AOD use is often seen as a risk factor for future revictimization. For example, Han et al. (2013, p. 2514) found that “alcohol is an important risk factor for ASA [adult sexual assault] among lesbians.” Across the studies, there is an implication that AOD consumption is a risk factor making victim-survivors vulnerable to another future sexual assault. AOD use is often referred to as “hazardous” in these papers (see, e.g., Hughes et al., 2010; Hequembourg et al., 2015; Wilkerson et al., 2021). Thus, it may seem that sexual violence victimization is directly caused by AOD use rather than considering the many and varied factors underpinning sexual violence.

Another trend was for papers to examine the interrelationship between AOD and CSA as potential risk factors for adult victimization (*n* = 16). Studies found that AOD use may be higher in those who experienced CSA, and this could be a risk factor in victimization as an adult (e.g., Balsam et al., 2011; Gilmore et al., 2014; Hughes et al., 2001). The studies in the review suggest a relationship between CSA, AOD use, and adult victimization. As our review has not looked at the broad literature on CSA, our analysis here warrants some caution. However, it again appears that AOD use is constructed as an individual-level risk factor for experiencing violence, at the expense of broader sociostructural drivers.

### Homophobia, Biphobia, Transphobia, and Minority Stress

Twenty-two papers examined the relationship between minority stress and discrimination and sexual violence victimization. Many also drew on minority stress as an explanatory framework for understanding the impacts of sexual violence. While the impacts of sexual violence are not the primary focus of this section, some of the papers understood minority stress/discrimination as both a risk factor *and* an impact of sexual violence, meaning it was at times difficult to disentangle these two issues (e.g., Kammer-Kerwick et al., 2019). Minority stress refers to “psychosocial stress derived from minority status” (Meyer, 1995, p. 38). In other words, members of minority communities routinely encounter stigmatizing or prejudicial attitudes, micro-aggression, and social exclusion, in turn contributing to ongoing stress (and distress) that can manifest in poor mental health and psychological well-being. In the context of our review, minority stress may arise from routinely encountering homophobia, biphobia, and transphobia, which themselves stem from cisnormativity and heteronormativity. While the concept of minority stress has been subject to critique, particularly in relation to its deficit-based approach (Frost & Meyer, 2023), in the context of our review, we are more concerned with examining how this concept has been taken up in relation to sexual violence, rather than debating the conceptual merits of minority stress itself.

Studies differed somewhat in how they defined and measured minority stress, meaning a lack of consistency across the studies. Some studies included external factors and/or the relationship between external discrimination and internalized stigma (e.g., previous negative experience of reporting, experiencing discrimination on the basis of their gender or sexual identity; e.g., Flanders et al., 2020; Jackson et al., 2017; Salim et al., 2020), while others focused on internal factors (e.g., internalized shame around sexuality or gender; e.g., Gold et al., 2009; Hequembourg et al., 2015; Murchison et al., 2017). While minority stress can lend itself to a structural analysis (e.g., structural homophobia), most of the studies included in our sample deployed this concept to look at individual behavior. The papers, in general, showed that there was a link between experiencing minority stress and experiencing sexual violence (e.g., Flanders et al., 2020; Murchison et al., 2017; Sabidó et al., 2015; Salim, 2020). Sabidó et al.’s (2015) study of Brazilian MSM, for example, found that experiencing self-defined homophobic discrimination in the past 12 months was the strongest predictor of participants having experienced sexual violence. Importantly, these authors are one of the rare examples that locate homophobic discrimination as structural and advocate for the need for legal and institutional change in Brazil. Beckman et al. (2018) also found that transgender military veterans who experienced sexual assault in the military experienced higher levels of “military minority stress,” PTSD, depression, and suicidal ideation compared to veterans who had not experienced military sexual assault. The authors theorize that minority stress may be a function of the masculine culture of the military, which contributes to the ostracization of gender non-conforming people, which could be a risk factor for sexual assault and revictimization. Conversely, Murchison et al. (2017, p. 227) found that internalized homophobia was associated with “any unwanted sexual experience and coercion,” but not sexual assault. Hequembourg et al. (2015) did not find any association between internalized homophobia and experiencing recent “severe” adult sexual assault for gay and bisexual men.

As noted, some studies did attempt to locate minority stress within a broader structural, social, and cultural context, and advocated the need to address these higher-level drivers of sexual violence against LGBTQ+ people. For example, in their study of bisexual young people’s experiences of sexual violence, Flanders et al. (2020) advocate for locating bisexual stigma (both internal and external) within an intersectional frame that considers issues such as incarceration or racism, which they argue must be addressed together. Otherwise, there was a general lack of critical examination of the structural causes of minority stress or internalized homophobia and sexual assault (Gold et al., 2009; Nightingale, 2021; Solomon et al., 2021). Rather, some papers tended to view these as individual pathologies that need to be overcome through psychological support or behavioral change. Murchison et al. (2017, p. 232), for example, suggest that experiencing minority stress may “reduce the likelihood of assertive responses to threat, increasing the chance of a completed assault” and that “internalized homophobia may be the primary cause of the abuse” (p. 233)—though they do acknowledge that some perpetrators may actively target individuals with internalized homophobia. By extension, there was an unspoken, if unintentional, implication that some responsibility could be attributed to the person for having minority stress which made them vulnerable to sexual assault. We recognize that understanding the relationship between internalized homophobia and sexual violence at an individual level may be important for informing therapeutic interventions (e.g., working with an individual to reduce internalized homophobia/stigma). However, in the absence of any acknowledgment of homophobia as a structural issue, or calls to tackle homophobia more broadly, statements such as these implicitly (if, again, unintentionally) run the risk of suggesting that the “problem” that needs to be tackled is an individual’s “sensitivity” to homophobia or inability to be assertive, rather than homophobia itself. It is clear that internalized stigma does negatively impact people’s lives and shapes experiences of sexual violence—though we would like to see a consideration of whether those who are able to articulate their experiences of minority stress are also better able to label their experiences of sexual violence. However, the attention to this as an individual rather than a structural issue could lead to an undue emphasis on individual behaviors and inadvertently place blame on victim-survivors.

### “Risky” Sexual Behavior and HIV Transmission

Twenty-three articles focused on “risky” sexual behavior as a risk factor for sexual violence, examining the relationship between experiences of sexual violence and subsequent engagement in “risky” sex (e.g., Wells et al., 2016), or looked at sexual practices that put people at risk of HIV/AIDS. Risky sexual behavior was often framed as having many sexual partners, engaging in sex work, drug use during sex, or not practicing safer sex (typically in relation to condom use). One study was focused on attitudes and 22 studies examined relationships between “risky” sexual practices and victimization or its relationship to HIV. Tilley et al. (2020) analyzed data from campus climate surveys from 10 U.S.-based higher education institutions, which examined survey items relating to peer norms in addition to experiences of sexual violence. The authors reported that LGBTQ+ participants reported higher rates of what they term “negative peer norms,” which they describe as “friends who would approve of engaging in risky sexual behavior” (Tilley et al., 2020, p. 66). They assessed whether having friends who approve of risky sexual behavior leads to higher rates of sexual victimization, and found higher rates of self-reported sexual violence for LGBTQ+ participants whose friends “approve of engaging in risky sexual behaviors” (Tilley et al., 2020, p. 68). However, they give limited detail on what constitutes “risky” sexual behavior, whether these measures are specifically tailored to LGBTQ+ students, or what role peer norms may play in facilitating or causing sexual violence.

The 22 studies examining “risky” sex and victimization followed this trend. Studies also tended to conflate different risk factors, and it was not always clear that risk factors necessarily had a causal role in sexual violence occurring. For example, when victim-survivors had also used alcohol or other drugs, it was difficult to determine what role intoxication played in the sexual assault, or what role working in the sex industry played compared to other forms of “risky” sex. Indeed, many of the studies that included risky sex and sex work tended to ignore other forms of inequality, such as poverty and the difficulties of working in a highly stigmatized profession. In general, risk was framed as an individual failure rather than considering how structural inequality could lead to disadvantage.

Further, several of these papers had what we interpreted as moralistic overtones. For example, one paper promoted the idea that being religious—and thus supposedly not engaging in sex outside marriage—was a protective factor against sexual assault (McGraw et al., 2020). A further central concern is that there is limited information on perpetration, and the contextual tools and resources that perpetrators draw on to aid their behavior. It is, therefore, difficult to draw conclusions about how such “risky” sexual behavior is associated with sexual assault. However, there were examples of articles that looked at those who engaged in “promiscuous” sex that considered the nuances of sexual practices in LGBTIQ+ communities (e.g., Braun, Terry, et al. 2009; Gaspar et al., 2021; Gavey et al., 2009). For example, some studies (e.g., Braun, Schmidt, et al., 2009) examined how norms of masculinity and sexual interaction in the gay male community produce a series of discourses that can lead to positive sexual experiences but can also work to normalize and excuse sexual violence, without suggesting that “risky” sex itself causes sexual violence, or that it is the responsibility of individuals to manage their risk of sexual violence.

In general, the definition of “risky sexual behavior” was unclear across the studies, beyond such descriptions as “sexual compulsivity” (Wells, 2016) or “hooking up” (McGraw et al., 2020). In part, it appeared that some studies consider sexual interaction outside of monogamy (i.e., having casual sex) to in some way constitute risky sexual behavior and as therefore accompanied by a higher risk of experiencing sexual assault (e.g., McGraw et al., 2020; Jaffe et al., 2020). Similarly, condomless penetrative sex was often constructed as inherently “risky,” and there was rarely exploration of whether participants engaged in other sexual health strategies to mitigate the likelihood of unwanted pregnancy or sexually transmitted infections (STIs)/human immunodeficiency virus (HIV) (e.g., using pre-exposure prophylaxis (PREP)/post-exposure prophylaxis (PEP), regular STI testing, using other forms of birth control and STI prevention, and so forth). While perhaps unintentional, these studies frequently framed sexual violence and “risky” sex in individualized ways that emphasized the behavior of victim-survivors, rather than the choices of perpetrators and sexual partners, or situating these experiences within broader sociostructural contexts. For example, Wells et al.’s (2016, pp. 3387–3388) U.S.-based study suggests that “associations with sexual victimization and risk arise as a function of partner selection,” with victim-survivors having “a higher tolerance for abusive and coercive behavior and . . . come to expect some level of coercion.”

Eleven papers focused on the relationship between sexual assault and/or sexual coercion and the risk for HIV transmission. While HIV can be an impact of sexual violence, the framing was often around sexual violence itself being a risk for contracting HIV, and thus, the person experiencing sexual violence was seen as a risk factor for others contracting HIV. Two papers found that there was higher HIV prevalence among those who experienced sexual violence (Shaw et al., 2012; Sabidó et al., 2015). Other papers considered sexual assault as a risk factor for HIV transmission due to the fact that having experienced sexual assault was associated generally with unprotected sex (e.g., Toro-Alfonso & Rodríguez-Madera, 2004) or the assaults themselves were more likely to involve condomless penetration (e.g., Kalichman et al., 2001). Discursively, these papers often worked to construct MSM who have experienced sexual assault or coercion as “risky” because they pose an increased risk for HIV transmission. This was explained plainly by Kalichman and Rompa (1995) who said that MSM who have experienced sexual coercion are “less inclined to act to protect themselves against HIV infection” and they represent a “public health problem” (p. 49). Similar attitudes continues today, as more recent papers still frame sexual violence prevention as important among MSM because it will prevent HIV (e.g., Hershow et al., 2021; Semple et al., 2017). Again, we do not intend to suggest that it is inherently problematic to consider the relationship between sexual violence and HIV transmission, nor with examining the intersection between preventing sexual violence and preventing HIV transmission—clearly, these are worthy endeavors. Our concern is more so with what we interpret as an individualizing and stigmatizing framing of both HIV and sexual violence across these papers, with both often constructed as the result of “risky” individuals engaging in “risky” practices.

### Child Sexual Abuse

As noted, the focus of our study is research that had at least 50% focus on *adult* LGBTQ+ sexual violence. The focus of our study was not CSA. However, within the studies that match the inclusion criteria, 29 studies included CSA victimization as a risk factor for experiencing sexual violence in adulthood, with studies spanning all sexualities and genders. In the context of our study, we are not seeking to provide a definitive overview of research into childhood sexual abuse experienced by LGBTQ+ communities. Rather, we include studies where CSA was included as a risk factor within a larger study of adult LGBTQ+ sexual violence.

The age range for childhood sexual abuse differed across the studies with some including adolescent sexual abuse (14–18 years old) with childhood sexual abuse (0–14 years old). Studies found a link between childhood sexual abuse and adult sexual abuse, with some framing childhood sexual abuse as a risk factor for adult sexual abuse (e.g., Hequembourg et al., 2011; Krahé et al., 2001). The majority of studies (*n* = 19) included LGB+ women, and nine of these compared heterosexual and LGB+ women, consistently finding that LGB+ women were more likely to have histories of childhood sexual abuse than heterosexual women. For example, in their U.S.-based study of 871 participants, including lesbians (*n* = 322), gay men (*n* = 214), and heterosexual women (*n* = 335), Balsam et al. (2011) found that lesbians had a higher rate (44%) of childhood sexual abuse compared to heterosexual women (31%) and gay men (30%). However, Han et al.’s (2013) U.S.-based study found that childhood sexual abuse was a predictor for adult sexual abuse for gay men but not necessarily for lesbians. They did not ask about the sexuality or gender of the perpetrator.

Overwhelmingly, studies focused on childhood sexual abuse as an individual-level risk factor, which was often analyzed alongside other risk factors such as binge drinking. There was limited structural analysis of childhood sexual abuse, despite research indicating that economic disadvantage, for example, is a structural risk factor for childhood sexual abuse (Doidge et al., 2017) and victimization in adulthood (Corbett et al., 2023). That is to say, CSA is a complex problem that is inadequately addressed in much of the research beyond the individual aspect of “risk.” Therefore, it is unclear whether CSA is a risk factor for adult sexual abuse or whether it is the other structural factors which underpin both child sexual and adult sexual violence. In other words, rather than looking at individual factors of CSA and adult sexual abuse, an analysis of structural factors could shed light on this co-occurrence.

## Discussion

This paper comprises one of two papers reporting on a critical scoping review of 108 empirical studies on adult LGBTQ+ people’s experiences of sexual violence. In the first paper (Hindes et al., 2025), we reported on the prevalence of victimization and perpetration, and in this paper we report on the risk factors associated with sexual violence. As noted in the companion paper, the review draws together a rapidly expanding field, and it is therefore timely to provide insights into the state of the field of LGBTQ+ sexual violence research. While the studies have added important LGBTQ+ perspectives to feminist research on sexual violence, we also found some significant issues, gaps, and limitations to the body of research.

Critiques of Individual-Level Risk Factors

Across the studies reviewed, we have raised a concern with the oversaturation of U.S. studies, and therefore, any results must be taken with caution as they may not reflect LGBTQ+ people’s experiences of sexual victimization outside of the U.S. We also raise significant concerns with the framing of much of the research. One particularly notable trend was the tendency for studies to examine LGBTQ+ sexual violence in relation to individual-level risk factors associated with sexual violence, including AOD use, minority stress, “risky” sexual behavior, and CSA. To be clear, we are not suggesting that it is automatically or always inherently problematic to consider how individual-level factors may increase the risk of experiencing sexual violence. However, across the sample, this focus created a meta-narrative of LGBTQ+ sex and LGBTQ+ lives as “risky” and requiring intervention, without locating these factors within broader contexts of heteronormativity and cisnormativity. For example, Krahé and Berger (2013, p. 399) concluded that bisexual participants’ higher rates of sexual victimization was because “engaging in both heterosexual and same-sex contact is indicative of a more active sexual life-style associated with greater vulnerability for sexual victimization.” Despite their finding that the majority of bisexual women’s experiences involved a male perpetrator, they still conclude this is due to their “active sexual lifestyle,” rather than experiences of biphobia, gendered power inequality, or other structural and social drivers of sexual violence.

Further, in this framing of “risky lifestyle,” there was limited focus on the perpetrator(s), which was a trend of much of the risk-focused research. Indeed, in the field of research on sexual violence in heterosexual contexts, while individualistic framings of risk for women victim-survivors persist, a significant body of work also focuses on risk factors for perpetration (Davis et al., 2018; Tharp et al., 2016). However, the almost exclusive focus on risk factors for victimization in LGBTQ+ contexts paints a narrative across the research of LGBTQ+ lives as risky. By extension, studies often (though not exclusively) emphasized interventions that could occur with victim-survivors or “at-risk” groups, rather than aiming to disrupt the structural power relations and norms that underpin sexual violence. Though perhaps inadvertent, this serves to responsibilize victim-survivors for preventing sexual violence and fails to ask *how* perpetrators might utilize these various contextual factors in enacting sexual violence. A focus on casual sex and hook-ups as “high risk” settings for sexual violence often implied that monogamous, long-term relationships were comparative sites of safety or were “low risk” for sexual violence, despite evidence that sexual violence often occurs in the context of intimate relationships (Tarzia, 2021; with the caveat that we excluded papers exclusively on intimate partner violence; however, very few papers were rejected on this basis, and very few papers looked at partner sexual violence in LGBTQ+ relationships, another underresearched topic). This framing also tends to imply that non-monogamous sex is itself inherently “risky,” rather than considering how this risk is actively produced through discourses that, for example, lays blame with victim-survivors for being sexually “promiscuous” or consuming AODs. Such blame was at times extended to HIV risk and had troubling stigmatizing overtones. CSA was similarly positioned as increasing the “risk” of experiencing adult sexual violence, with limited consideration of structural drivers, or how previous experiences of abuse may be drawn on to position victim-survivors as vulnerable to further violence.

While most studies did not adopt an intersectional lens, those that did highlight how experiencing compounding forms of inequality could impact sexual violence. Again, some studies tended to position victim-survivors as “risky” individuals whose poor lifestyle choices (such as the consumption of AODs) or internalized homophobia amplified the risk of experiencing sexual violence. As we have stressed throughout, while there is a place for work examining how individual-level factors shape the harms and impacts of sexual violence, we urge researchers working in this field to reflect on how such framings may work to responsibilize or blame victim-survivors, and to ensure that victim-survivors experiences are carefully contextualized within a broader structural framing of sexual violence.

### Research Gaps

Finally, our review has identified clear and significant gaps in the literature. First, research in this field has predominantly been undertaken in the U.S., and therefore, there is an urgent need for research on LGBTQ+ sexual violence across a more diverse range of geographic, social, and cultural locations. Second, and by extension, the context of the research on LGBTQ+ sexual violence often focuses on, or is in response to, political and public health issues, for example, campus sexual assault, HIV infection, military sexual assault, and “hazardous” drinking. These contexts often shaped how data were collected and analyzed, making it difficult to know how the findings related to LGBTQ+ sexual violence more broadly outside of these specific political and public health issues. We also reiterate the limited scope of the field which rarely extends beyond the individual risk factors to consider the broader sociocultural context and structural hierarchies beyond gender (such as capitalism, colonialism, heterosexism, or cis-sexism) that shape LGBTQ+ people’s experiences of violence beyond their individual level of control (Donovan & Barnes, 2020).

Third, there were also significant gaps in who has been the subject of study. For example, there are limited examples of people who experience state-sanctioned sexual violence other than four studies with formally incarcerated people (Jenness et al., 2019; Jenness & Sexton, 2021; Ratkalkar & Atkin-Plunk, 2020; Wilson et al., 2017). There are no studies that look at institutional sexual violence, such as in aged care (Australian Institute of Family Studies, 2022). Research with an intersectional focus is also limited, particularly due to the focus on North American college cohorts—though there are important exceptions to this (e.g., Ussher et al., 2020). As we also raise in the accompanying paper (Hindes et al., 2025), many studies only focused on LGB communities—thus excluding the experiences of transgender, gender diverse, and diverse sexualities (e.g., pansexual)—or collapsed the diverse experiences of LGBTQ+ communities into a singular category of analysis. The collapsing of identities continued to be an issue across the studies analyzed here, and it is important that future research aims to account for the specificities of experiences within and across the diverse LGBTQ+ umbrella.

Fourth, research to date has not adequately captured the factors associated with experiencing sexual violence from a queer perspective. This is, arguably, at least in part due to the apparent lack of attention across studies to heteronormativity and cisnormativity and how they shape experiences of sexual violence. As we discuss in-depth in our accompanying paper (Hindes et al., 2025), it was common for studies to draw on scales that had originally been designed to capture the experiences of cisgender, heterosexual women, and it is currently unclear to what extent these measurement tools accurately reflect LGBTQ+ people’s experiences. Research on specific forms of violence experienced by LGBTQ+ people has, to date, focused on family violence, and it has shown LGBTQ+ people face additional factors such as threats of outing (Carman et al., 2020; LGBTIQ Domestic and Family Violence Interagency and Centre for Social Research in Health, 2014). Future research is needed to map the specific forms of sexual violence experienced by LGBTQ+ people and to ensure that (particularly) quantitative studies are less likely to underrepresent or occlude the nature and extent of, and the contextual factors underpinning, LGBTQ+ victim-survivors’ experiences.

In saying all of this, we do not wish to detract from the important research undertaken by feminists and LGBTQ+ sexual violence researchers. Rather, we argue that an expanded approach to LGBTQ+ sexual violence research could make significant contributions to the field. For example, queer theory could be drawn on to ensure that empirical studies are situated within a theoretical framework that centers the disruption of heteronormativity and cisnormativity, as well as other forms of intersecting inequality. To date, sexual violence research adopting a queer theoretical approach has considered how relationships can be reconceptualized beyond just monogamous couples (Ison, 2019), how sexual negotiation can be understood beyond heteronormative consent frameworks (Hindes, 2024; Webber et al., 2024), elevated the voices of LGBTQ+ communities (Layard et al., 2022), and considered sexual violence within broader structures of inequality (Patterson, 2016; Ussher et al., 2020). It is also important that we look beyond seeing queer lives as “risky” and understand how LGBTQ+ gender and sexuality can be a site of joy and pleasure (Hindes, 2024; Wright et al., 2024). This is particularly important in light of the continued heteronormative understandings of gender-based violence (Donovan & Barnes, 2020) and the real-world implications this has on LGBTQ+ communities.

## Key Findings

• Key risk factors that are studied to understand LGBTQ+ sexual violence include AOD use, experiences of minority stress and discrimination, engaging in “risky” sexual behavior, and previous instances of CSA. These risk factors are often examined through an individualistic lens, focusing on how a survivor’s behaviors or experiences contributed to their victimization and how individual behavior change can lessen these experiences.
• AOD use was shown to be a risk factor for sexual violence, yet the studies often only assessed recent AOD use (e.g., last month) and adult sexual violence victimization (e.g., sexual assault since 14) to claim that LGBTQ+ victim-survivors had high rates of AOD use. However, it was not always clear that there was a causal relationship between these factors, given the disparate timeline and lack of consideration of other life factors.
• Minority stress was often conceived of as an individual issue which contributed to why an LGBTQ+ person was sexually assaulted. As a result of this individual-level focus, there were undertones of victim-blaming, however unintentional, if a person had high levels of minority stress or internalized shame before or after a sexual assault.
• “Risky” sexual behavior had diverse meanings across the studies, but often held troubling moralistic values on what constituted acceptable sexual behavior and, thus, how supposedly “risky” behavior could lead to a person being victimized. “Risky” sexual behavior that may lead to sexual violence victimization was also seen as a risk factor for HIV transmission.
• While our study was focused on adult sexual violence, within the sample, there was a focus on CSA as a risk factor for adult sexual violence. However, the focus was again on individual-level risk factors for CSA rather than situating this within broader structural understandings of CSA and how it relates to adult sexual violence.

## Implications for Research, Policy, and Practice

• **Community engaged research:** While individual-level research is not inherently or always problematic, researchers should carefully consider the implications of focusing on these factors, particularly in LGBTQ+ people’s lives, which are often already stigmatized. Future research must engage with LGBTQ+ communities to design research approaches that do not cause harm.
• **Sociocultural contexts:** Current research tends to take an individualistic approach that frames LGBTQ+ sexual violence in terms of discrete risk factors, rather than situating it within a broader structural and sociopolitical context. Future research must go beyond individual-level risk factors to consider broader sociocultural contexts.
• **LGBTQ+ specific approach:** Where it is relevant to study individual-level risk factors, greater attention to nuances is needed to ensure that research addresses the specific risk factors for LGBTQ+ people.

## Conclusions and Limitations

In this paper, we have critically examined the state of existing research relating to LGBTQ+ sexual violence, specifically the risk factors associated with victimization. Together with our accompanying review paper on victimization and perpetration prevalence (Hindes et al., 2025), these contributions offer a comprehensive overview of the literature and the state of the field of LGBTQ+ adult sexual violence. However, there are some limitations to the review that should be noted. Our review synthesized academic peer-reviewed literature, and therefore, important non-peer-reviewed literature has not been included. The scoping review had a global focus, but was limited to English language papers due to authors’ language limitations. While we undertook a comprehensive search of the literature, with expansive search terms, it is possible that some papers may have been excluded due to differing terminology. Overall, our contribution in this paper is to demonstrate the oversaturation of U.S. studies, methodological limitations of research to date, lack of attention to risk factors beyond the individual level, and a meta-narrative across papers that discursively constructs queer lives as “risky” and in need of management and intervention. We call for future research to expand the field beyond heteronormative and cisnormative inquiry.

## Supplemental Material

sj-docx-1-tva-10.1177_15248380241311930 – Supplemental material for LGBTQ+ Adult Sexual Violence Critical Scoping Review: Victimization Risk Factors Supplemental material, sj-docx-1-tva-10.1177_15248380241311930 for LGBTQ+ Adult Sexual Violence Critical Scoping Review: Victimization Risk Factors by Jessica Ison, Sophie Hindes and Bianca Fileborn in Trauma, Violence, & Abuse

sj-docx-2-tva-10.1177_15248380241311930 – Supplemental material for LGBTQ+ Adult Sexual Violence Critical Scoping Review: Victimization Risk Factors Supplemental material, sj-docx-2-tva-10.1177_15248380241311930 for LGBTQ+ Adult Sexual Violence Critical Scoping Review: Victimization Risk Factors by Jessica Ison, Sophie Hindes and Bianca Fileborn in Trauma, Violence, & Abuse
